# 

**Published:** 1857-02

**Authors:** 


### RECEIPTS ON SUBSCRIPTIONS,

### Up to Feb. 1st, 1857.

### ILLINOIS.

| | | |
|---|---|---|
| L. D. Smedley, | Vol. 6, | $2.00 |
| M. Davis, | Vol. 5 & 6, | 4.00 |
| H. R. Payne, | Vol. 6, | 2.00 |
| P. W. Hill, | " 6, | 2.00 |
| D. T. Morgan, | " 6, | 2.00 |
| W. W. Allport, | " 6, | 2.00 |
| Owen Wright, | " 6, | 2.00 |
| J. W. Trabue, | " 6, | 2.00 |
| C. Goodbrake, | " 6, | 2.00 |
| E. R. Boardman, | " 6, | 2.00 |
| —— Mergler, | " 6, | 2.00 |
| —— Hoffman, | " 5, | 2.00 |
| F. R. Payne, | " 5, | 2.00 |
| R. E. Rich. | " 5, | 2.00 |
| J. M. Sudduth, | Vol. 4 & 5, | 4.00 |
| Orrin Peak, | Vol. 6, | 2.00 |
| T. G. Vandever, | " 6, | 2.00 |
| G. W. Cornwell, | " 6, | 2.00 |
| T. D. Fisher, | " 6, | 2.00 |
| L. C. White, | " 4, | 2.00 |
| J. B. Glass, | " 6, | 2.00 |
| Hosea Davis, | " 5, | 2.00 |
| J. D. Cope, | " 6, | 2.00 |
| E. E. Webster. | " 6, | 2.00 |
| A. S. Haskell, | " 5, | 2.00 |
| L. A. Winslow, | Vol. 4,5,6, | 6.00 |

### ILLINOIS—Continued.

| | | |
|---|---|---|
| S. T. Trowbridge, | Vol. 3,4, | 4.00 |
| G. M. Harrison | Vol. 5, | 2.00 |
| J. Blount, | " 6, | 2.00 |
| D. W. Stormont, | " 6, | 2.00 |
| J. M. Steele, | " 6, | 2.00 |
| T. W. Floyd, | " 6, | 2.00 |

### MINNESOTA.

| | | |
|---|---|---|
| O. Allen, | Vol. 6, | 2.00 |

### INDIANA.

| | | |
|---|---|---|
| W. M. Denny, | Vol. 6, | 2.00 |
| S. W. Ritchey, | " 5, | 2.00 |
| H. B. Howes, | Vol. 4 & 5, | 4.00 |
| D. Hutchinson, | " 4 & 5, | 4.00 |
| M. H. Bonnel, | Vol. 6, | 2.00 |
| Long & Ellis, | " 6, | 2.00 |
| P. Myer, | Vol. 5 & 6, | 4.00 |
| S. C. Gray, | Vol. 5, | 2.00 |
| A. G. Standeford, | " 5, | 2.00 |
| T. P. Seller, | " 5, | 2.00 |
| H. Duncan, | " 5, | 2.00 |
| D. W. Taylor, | " 6, | 2.00 |
| J. Adrain, | " 6, | 2.00 |
| Robert Winton, | " 6, | 2.00 |
| E. Jenison, | " 5, | 2.00 |

### MICHIGAN.

| | | |
|---|---|---|
| Chas. Shepherd, | Vol. 4&5, | 4.00 |
| J. W. Hall, | Vol. 5, | 2.00 |
| Edwin Stewart, | Vol. 5&6, | 4.00 |

### WISCONSIN.

| | | |
|---|---|---|
| Clark & Rice, | Vol. 5, | 2.00 |
| W. & A. Gordon, | " 5, | 2.00 |
| O. D. Coleman, | " 5, | 2.00 |
| A. P. Adams, | Vol. 5 & 6, | 4.00 |
| James Cady, | Vol. 5, | 2.00 |
| Wm. Carger, | " 6, | 2.00 |
| Benj. Durham, | " 6, | 2.00 |
| W. H. Walker, | Vol. 4&5, | 4.00 |
| Rowley Morris, | " 5&6, | 4.00 |

### IOWA.

| | | |
|---|---|---|
| P. M. Kerlin, | Vol. 5, | 2.00 |
| J. H. Bowers, | " 6, | 2.00 |
| M. A. Dashiel, | " 4, | 2.00 |
| John McHugh, | " 5, | 2.00 |
| J. J. Morgan, | [illegible] | 2. |
| F. M. Brady, | Vol. 4 & 5 | 4.0 |
| H. C. Rawson, | Vol. 6, | 2.0 |

